# Case Report: Cotard’s syndrome associated with suicide attempt-related delirium

**DOI:** 10.3389/fpsyt.2026.1890939

**Published:** 2026-07-03

**Authors:** Richárd Flach, Júlia Éva Varga, Róbert Herold, Vita Bányavölgyi, Péter Osvath, Sándor Fekete, Viktor Voros, Tamás Tényi

**Affiliations:** Department of Psychiatry and Psychotherapy, Medical School, University of Pécs, Pécs, Hungary

**Keywords:** Cotard’s syndrome, late-life psychosis, mild cognitive impairment, nihilistic delusions, suicide attempt

## Abstract

Cotard’s syndrome is a rare psychopathological phenomenon characterized by nihilistic delusions concerning one’s body, existence, or death. We report the case of an 88-year-old man with no previous psychiatric treatment who was admitted following a medication-overdose suicide attempt in the context of a severe depressive crisis. After intensive and internal medical treatment, he developed a fluctuating confusional state characterized by disturbances of orientation, impaired attention, paranoid ideation, and nihilistic beliefs. During this period, he repeatedly stated that he was dead, had been cremated, and that his wife had also died. Notably, these nihilistic beliefs persisted during relatively lucid intervals, even as the severity of the disturbance of consciousness fluctuated. Severe depressive symptoms, somatic decompensation, metabolic abnormalities, sensory impairment, frontotemporal atrophy, and mild neurocognitive impairment were present. The clinical picture was interpreted as Cotard-type nihilistic delusions emerging during a fluctuating delirious state in the context of severe depression, suicidal behavior, and underlying neurocognitive vulnerability. Treatment with risperidone, mirtazapine, somatic stabilization, and supportive psychotherapy was followed by gradual resolution of both the confusional state and the nihilistic delusions. This case highlights the complex interplay between delirium, severe depression, suicidal behavior, and neurocognitive impairment in the development of Cotard-type phenomena and underscores the importance of a careful differential diagnostic approach when nihilistic delusions arise in medically and cognitively vulnerable older adults.

## 1 Introduction

Cotard’s syndrome (CS), originally described by Jules Cotard in 1882 as *délire de négation*, is a rare psychopathological phenomenon characterized by nihilistic delusions concerning one’s body, existence, or death (1, 2). Although historically regarded as a distinct psychiatric disorder, contemporary authors generally conceptualize CS as a phenomenological syndrome that may occur in the context of various psychiatric, neurological, and medical conditions rather than as an independent nosological entity (3, 4). Interestingly, Cotard’s influence extended beyond medicine into literature, as Marcel Proust is thought to have partly modeled the character of Professor Cottard in *In Search of Lost Time* on the French neurologist and psychiatrist Jules Cotard (5).

The clinical presentation of CS is heterogeneous and ranges from beliefs involving the loss of bodily or mental functions to the complete denial of one’s own existence or that of the external world (6). Reviewing the original work of Jules Cotard, Cousin et al. (7) emphasized that patients may progressively deny the existence of their bodies, organs, and ultimately their entire existence. Classical descriptions suggest that Cotard syndrome may be preceded by a prodromal phase characterized by diffuse anxiety, irritability, and depressive symptoms (6). While neither the DSM-5 (8) nor the ICD-11 (9) recognizes CS as a distinct diagnosis, its clinical significance remains considerable because it is associated with severe psychopathology, impaired reality testing, self-neglect, treatment resistance, and an elevated risk of suicide (10, 11). Although precise epidemiological data remain limited, the prevalence of CS in adult populations has been estimated to range between 0.57% and 3.2% (11).

CS is most commonly reported in association with severe depressive disorders, but it has also been described in schizophrenia spectrum disorders, delirium, neurocognitive disorders, and a broad range of neurological and medical conditions (1, 3, 12, 13) In their classic review of 100 published cases, Berrios & Luque (10) found depressive symptoms in 89% of patients, while nihilistic delusions concerning the body and existence were present in 86% and 69% of cases, respectively. Anxiety, guilt, affective instability, hypochondriacal concerns, perceptual disturbances, and suicidal behaviour were also frequently reported. Similarly, Solimine et al. (14) highlighted the close association of CS with severe depression, anxiety, guilt, and hypochondriacal phenomena, whereas Kreczyńska et al. (13) emphasized the heterogeneous nature of the syndrome and the frequent coexistence of affective, perceptual, and psychotic symptoms. López (15) further noted that the clinical course and prognosis of Cotard phenomena may vary considerably depending on the underlying psychopathological condition.

Suicidal behaviour represents one of the most clinically relevant aspects of CS. Although precise suicide rates are unknown, several case reports suggest a substantial risk of self-harm and self-neglect. Bosco et al. (11) emphasized that food refusal and self-starvation associated with nihilistic delusions may be misinterpreted as eating disorders despite being manifestations of a potentially life-threatening psychiatric condition. Similarly, Grover et al. (16) reported that severe nutritional compromise, refusal to eat, and, in rare cases, catatonic symptoms may complicate the clinical presentation.

Current neurobiological models propose that Cotard syndrome may be associated with dysfunction within distributed frontotemporal, parietal, and limbic networks involved in self-representation, body awareness, emotional processing, and reality monitoring; however, the available evidence remains limited and is based largely on case reports and small neuroimaging studies (17, 18). According to Dieguez (17), impairments in self-perception, body representation, emotional processing, and right-hemispheric functioning may contribute to the emergence of nihilistic delusions. Neuroimaging studies have suggested an association between CS and structural or functional abnormalities involving frontotemporal regions and, in some cases, the non-dominant hemisphere (17, 19). In a series of 12 patients, Sahoo and Josephs (19) reported frontal lobe abnormalities, generalized cerebral atrophy, ischemic lesions, and right- or bilateral hemispheric involvement, supporting the notion that CS frequently occurs in the context of underlying neurological pathology.

Consistent with its heterogeneous etiology, treatment is directed toward the underlying disorder. Reported treatments have included antidepressants, antipsychotics, mood stabilizers, electroconvulsive therapy (ECT), and management of underlying medical conditions (1, 3). ECT remains one of the most consistently effective interventions, particularly in severe affective presentations (3). Nevertheless, Bistas & Mirza (20) emphasized that the pathophysiology of CS remains incompletely understood and that careful neurological and psychiatric evaluation remains essential for accurate diagnosis and treatment planning.

The present report describes an 88-year-old man who developed Cotard-type nihilistic delusions in the context of severe depression, suicidal behaviour, fluctuating disturbance of consciousness, somatic decompensation, and mild neurocognitive impairment. The case highlights the diagnostic challenges involved in distinguishing between delirium, affective psychopathology, and neurocognitive vulnerability in the development of Cotard-type phenomena in later life.

## 2 Case presentation

An 88-year-old male patient with no prior history of psychiatric treatment was admitted to the geriatric psychiatry ward following a suicide attempt by medication overdose. He ingested large quantities of antihypertensive and hypnotic medications (nebivolol, rilmenidine, doxazosin, amlodipine, and zolpidem) with the express intent of ending his life. He had an extensive medical history, including hypertension, type 2 diabetes mellitus, chronic atrial fibrillation, glaucoma, chronic renal insufficiency, microcytic anemia, and multiple previous abdominal surgeries, including a right hemicolectomy due to cecal adenocarcinoma. In recent months, he had been hospitalized multiple times for somatic care due to infectious diarrhea, anemia, and general physical decline, and his physical condition had gradually deteriorated. Following a suicide attempt, he required intensive care due to cardiovascular instability and metabolic abnormalities; after receiving internal medicine and cardiology treatment, he was transferred to our psychiatric ward due to a worsening of his mental state.

One month prior to this, he received outpatient care in the emergency department, where, according to the medical records, one week and then two days prior, he had again attempted to take his own life by ingesting medications (according to his account: nebivolol 5 mg 10 times, rilmenidine 1 mg 10 times, doxazosin 10 times, amlodipine 15 times, zolpidem 3 times); however, aside from significant weakness, he had no other complaints. Subsequently, he received outpatient care at our clinic to rule out a suicidal crisis. During her evaluation, he was calm and cooperative, fully oriented in all respects; he explained that he had argued with her daughter over the phone, so he told him, “My blood pressure is high anyway, so I’ll take more blood pressure medication.” He firmly denied any suicidal intentions or ideation. He listed all his internal medicine medications without omission. No psychotic symptoms were observed. He has many people helping him in his daily life; his son visits him every day, and the residents of the home help him with shopping and picking up his medications. Recently, he has been hospitalized several times, which he found difficult to cope with, and he was worried about his wife, who is being cared for by his daughter. They maintain contact by phone and online every day.

According to the patient’s daughter, who lives in another city, a neighbour took the patient’s medications—at the patient’s request—one month ago, after the patient had taken his medication but before the recent suicide attempt; that evening, the patient called his relatives to say that he had taken about thirty blood pressure pills. The relatives initially considered the incident a “fabrication, “ as the patient had repeatedly threatened to commit a similar act for years.

He was transferred from the Intensive Care Unit to the Department of Internal Mecicine for further stabilization of his physical condition, where he was found to be in satisfactory condition and oriented in time and place. During a clinical psychological evaluation, he stated that his wife had been living in another city for 14 months, and since he could not move with her due to limited living space, he was living alone. A few days ago was their 50th wedding anniversary, which they spent apart. Last week, he had a phone argument with his daughter, after which he was unable to reach her despite multiple calls. At that point, he felt he was only a burden to his family, which is why he took medication with suicidal intent. He continued to express feelings of worthlessness, and indirect suicidal intentions also emerged. During supportive patient care, her mood showed signs of improvement; he became more emotionally accessible, active, and open, and her mood approached a state of euthymia.

The following day, his condition worsened significantly; he developed delirium and paranoid delusions, required restraint, and was transferred to our clinic. Upon admission to the psychiatric ward, the examination was complicated by hearing and vision impairment (glaucoma simplex). The patient firmly asserted that he was dead, currently in the morgue, and would soon be transported to the crematorium. He reported that his wife had also died and that they had been transported to the hospital together. During the examination, he stated that he no longer had “even his bones, because they had been cremated”—he insisted on these statements with delusional confidence. He acknowledged the attempted suicide by medication, yet paradoxically emphasized that “that is not why he died.” The patient’s consciousness was alert, though fluctuating delirium was identifiable. He was disoriented as he was uncertain regarding his location in space and time. His thinking was slowed, his speech showed reduced productivity, and his content was dominated by explicit nihilistic delusions. He stated with conviction that he had died, his body had been cremated, and his wife was also dead. His mood was depressive, accompanied by anxiety and the recall of previous suicidal impulses. No perceptual disturbances were clearly observable. His behavior was tense but cooperative and controllable, and his psychomotor activity was slowed dowwn. Based on cognitive assessment, mild cognitive impairment was likely; however, the interpretation of cognitive performance was complicated by the patient’s recent recovery from an acute clinically diagnosed delirious state, characterized by fluctuating consciousness, impaired attention, and disorientation.

**Table 1** summarizes the events and clinical decision points leading up to admission to the geriopsychiatric ward.

**Table 1:** Timeline of clinical events and key diagnostic-therapeutic turning points prior to gerontopsychiatric admission.

Time point/phase	Event	Clinical significance
Months prior to admission 11-26.09.2025. and 09.10.2025.	Recurrent somatic hospitalizations (infection, anemia, general physical decline)	Increasing physical vulnerability and functional deterioration No psychiatric evaluation was carried out
~1 month earlier 15.10.2025.	Medication overdose with suicidal intent, emergency outpatient care	Suicidal crisis and ambivalent suicidal behavior
Outpatient psychiatric assessment 16.10.2025.	Oriented and cooperative state without psychotic symptoms; denial of suicidal intent	Temporary psychological compensation
Prior to repeated suicidal event 11.2025.	Family conflict, social isolation, increasing feelings of uselessness and being a burden to others	Deepening crisis state and depressive symptomatology
Suicide attempt 15.11.2025.	Ingestion of large quantities of antihypertensive and hypnotic medications	Active, potentially lethal suicidal act
Intensive care treatment 15-17.11.2025.	Treatment for cardiovascular instability and metabolic abnormalities	Acute somatic condition increasing risk of delirium
Internal Medicine 17-21.11.2025.	Initially oriented state, later emergence of indirect suicidal content	Fluctuating mental state, disturbance of consciousness
Re-exploration on the following day 21.11.2025.	Acute delirium, paranoid ideation, need for physical restraint	Delirious–psychotic decompensation psychiatric consultation
Admission to gerontopsychiatric ward 21.11.2025.	Nihilistic delusions (“he was dead, “ “he had been cremated”), delirious episode, symptoms characteristic of Cotard’s syndrome	Acute psychotic state requiring urgent psychiatric treatment

As summarized in **Table 1**, the nihilistic delusions were conceptualized as Cotard-type phenomena emerging within a complex clinical context involving severe depression, suicidal behaviour, somatic decompensation, fluctuating delirium, and underlying neurocognitive vulnerability. Retrospective reconstruction of the clinical course suggested that a major depressive episode had developed prior to the suicide attempts and likely contributed both to the suicidal crisis and to the subsequent emergence of nihilistic beliefs. Following the medically serious medication-overdose suicide attempt, the patient experienced significant somatic deterioration and a fluctuating disturbance of consciousness, which appeared to aggravate the clinical picture and facilitate the development and persistence of the delusional symptoms. Importantly, nihilistic beliefs were not confined to periods of overt delirium but were also observed during relatively lucid intervals, suggesting that the Cotard-type symptoms could not be explained solely by the delirious state.

## 3 Diagnostic assessment

Laboratory tests revealed hypokalemia, elevated inflammatory markers, transient renal dysfunction, and known microcytic anemia. A cranial CT scan performed according to the dementia protocol showed frontotemporal-predominant cerebral atrophy, with no other pathological intracranial abnormalities. Cognitive screening tests performed after resolution of the delirious state showed an MMSE score within the normal range (29/30); however, the Addenbrooke’s Cognitive Examination (ACE: 83/100) indicated mild cognitive impairment (MCI), with deficits particularly evident in verbal fluency and retrograde memory. The MMPI-2 results were interpreted cautiously, as the assessment was conducted during recovery from acute delirium and following a period of severe depressive symptomatology. Therefore, the profile was not considered a stable representation of personality structure but rather a reflection of the patient’s psychological state during a clinically vulnerable period. Within these limitations, the findings suggested significant psychological distress, elevated internal tension, depressive affect, reduced resilience, and vulnerability to suicidal ideation. Indicators of anxiety, cognitive disorganization, emotional withdrawal, and unusual internal experiences were interpreted as state-dependent phenomena. At the time of admission, the score on the Montgomery and Asberg Depression Rating Scale (MADRS) was 45 out of 60, indicating severe depression.

During the 14-day inpatient treatment in the geriatric psychiatry ward, we initiated pharmacotherapy with an antipsychotic (risperidone) and an antidepressant (mirtazapine), supplemented by individualized supportive psychotherapy. Risperidone was initially administered at a dose of 0.5 mg twice daily and was reduced to 0.5 mg in the evening from the fifth treatment day onward. Mirtazapine 30 mg/day was introduced on the fourth treatment day and continued throughout the remainder of the hospitalization. Pharmacological treatment was accompanied by ongoing medical management of the patient’s physical condition, somatic stabilization, restoration of the sleep–wake cycle, and supportive psychological interventions.The interventions focused on processing the crisis situation, strengthening reality testing, mobilizing personal resources, developing problem-solving skills, and supporting cooperation with treatment. Regular contact and psychoeducation were provided to family members. As a result of the treatment, the patient’s sleep-wake cycle normalized, his mood stabilized at a euthymic level, and his anxiety decreased. The emergence of nihilistic delusions coincided with the state of acute consciousness disturbance and was most markedly observable primarily in the early stages of delirious-psychic decompensation. The CS presumably emerged as part of a delirious state associated with acute somatic stress, metabolic abnormalities, mild cognitive impairment, and sensory deficits. At the same time, the affective nature of the symptoms—the presence of a pronounced depressive mood, guilt, feelings of worthlessness, and suicidal impulses—suggests that not only organic disturbance but also a severe affective crisis may have played a role in the development of nihilistic delusions.

During the first few days of inpatient psychiatric treatment, the delusions persisted intermittently; however, as the sleep-wake cycle normalized, somatic symptoms stabilized, and antipsychotic and antidepressant treatment took effect, they gradually subsided and disappeared. Suicidal intent was denied even after repeated inquiries. At discharge, no acute psychotic symptoms or suicidal impulses were observed, and his MADRS score had decreased to 19/60.

At the two-month follow-up, the patient’s general psychiatric condition remained stable. No active psychotic symptoms or suicidal ideation were observed, and he took his medication cooperatively. He retrospectively described the suicide attempt as a mistake and expressed a future-oriented attitude toward placement in a nursing home. However, cognitive decline and symptoms suggestive of a developing neurocognitive disorder became more prominent, particularly in the form of reduced spontaneity and more limited communication during periods.

## 4 Discussion

Berrios & Luque (10) classically identified three main subtypes of CS: psychotic depression, in which patients suffer from melancholia and nihilistic delusions; a “pure” Cotard Type I without depressive symptoms; and a mixed Cotard Type II, characterized by anxiety, auditory hallucinations, and suicidal behavior. Our case most closely resembles the psychotic depression subtype, as the patient exhibited a severe depressive state, supported by a MADRS score of 45, while marked nihilistic delusions and suicidal behavior were present. At the same time, the uniqueness of this case lies in the fact that the Cotard symptoms developed acutely following a non-fatal suicide attempt and transient somatic decompensation, against a background of significant organic pathology, which was also confirmed by the results of psychological examinations.

To clarify the temporal sequence of clinical events and address the relationship between depressive symptoms, suicidal behavior, somatic decompensation, delirium, Cotard-type nihilistic delusions, treatment, and outcome, we summarized the patient’s clinical course in **Table 2**. The timeline also provides additional details regarding the duration of inpatient treatment and the pharmacological interventions administered during hospitalization.

**Table 2:** Relationship between depressive symptoms, suicidal behavior, delirium, Cotard-type nihilistic delusions, treatment, and outcome.

Clinical phase	Major events and findings
Prodromal period	Progressive depressive symptoms, hopelessness, social withdrawal, reduced motivation, and increasing psychological distress.
Suicidal crisis	Development of suicidal ideation followed by a medication-overdose suicide attempt.
Second suicide attempt	A more severe medication-overdose suicide attempt resulting in hospital admission and acute medical complications.
Acute medical deterioration	Somatic decompensation and metabolic abnormalities requiring intensive and internal medical treatment.
Delirious state	Fluctuating disturbance of consciousness with disorientation, impaired attention, altered awareness, sleep-wake cycle disruption, and paranoid ideation.
Emergence of Cotard-type symptoms	During the period of fluctuating mental status, the patient repeatedly stated that he was dead, had been cremated, and that his wife had also died.
Persistence of nihilistic delusions	Importantly, nihilistic beliefs were not limited to periods of overt delirium and were also observed during relatively lucid intervals.
Neurocognitive assessment	Mild neurocognitive impairment was identified. Cranial CT demonstrated predominantly frontotemporal cerebral atrophy.
Psychiatric treatment	Risperidone 0.5 mg/day, mirtazapine 30 mg/day, somatic stabilization, supportive psychotherapy, restoration of sleep-wake rhythm, and family involvement.
Clinical improvement	Gradual resolution of delirious symptoms, improvement in reality testing, and progressive disappearance of nihilistic delusions.
Follow-up	No active psychotic symptoms or suicidal ideation. Cognitive difficulties remained noticeable, raising ongoing concerns regarding an evolving neurocognitive disorder.

The case presented is consistent in several respects with the observations of Oberndorfer et al. (21), who, in the context of the Cotard phenomenon associated with hypoactive delirium, emphasized the role of organic factors—particularly infections, electrolyte imbalances, and organ decompensation—in the development of nihilistic delusions. In our case, the onset of full-blown CS was preceded by severe depression, significant somatic vulnerability, and acute drug intoxication, suggesting that affective and organic factors may have jointly contributed to the development of the psychotic state. In older adults, the CS can easily be confused with delirium, dementia, or psychotic depression; therefore, a careful examination of the organic background is of paramount importance, all the more so because the improvement of psychotic symptoms in parallel with the resolution of the somatic condition reinforces the etiological role of organic factors. According to Enoch & Ball (6), within the psycho-organic group, symptoms and signs of an impairment of the sensorium such as disorientation, defects of grasp, attention and concentration, together with memory loss, may be present. If CS is associated with an organic condition, treatment is based on targeted therapy for the underlying disease. As acute, reversible somatic conditions—such as infections or toxic states—resolve, symptoms may improve significantly, whereas in cases of irreversible brain damage or dementia, the prognosis is less favorable. Thus, identifying the underlying organic cause is of critical importance for the outcome. These considerations are also relevant when evaluating possible underlying neurodegenerative disorders in older adults presenting with Cotard-type symptoms. Recent evidence suggests that late-onset suicidal behaviour may, in some cases, represent an early manifestation of neurodegenerative disease, particularly dementia with Lewy bodies (DLB) (22), while Cotard syndrome has also been reported in association with Parkinson’s disease psychosis (23). In light of these observations, DLB and PD were carefully considered during the diagnostic process in the present case. Several aspects of the presentation, including advanced age, fluctuating disturbance of consciousness, psychotic symptoms, cognitive impairment, and suicidal behaviour, were compatible with a possible neurodegenerative contribution. However, several clinical findings argued against a diagnosis of DLB or PD as the primary explanation for the patient’s presentation. Specifically, no spontaneous parkinsonian signs were observed during hospitalization, including resting tremor, rigidity, bradykinesia, or postural instability. Furthermore, the patient did not exhibit clinically significant neuroleptic sensitivity during treatment with risperidone, a phenomenon frequently reported in DLB. Neuropsychological assessment suggested mild cognitive impairment, while cranial CT performed as part of the dementia workup demonstrated predominantly frontotemporal cerebral atrophy rather than findings specifically suggestive of Lewy body disease. Importantly, we do not exclude the possibility that an underlying neurodegenerative process contributed to the patient’s vulnerability. However, based on the available clinical, neuropsychological, and neuroimaging findings, the evidence was considered insufficient to establish a diagnosis of DLB or PD.

Our case can be compared in several respects to the Cotard case reported by Moschopoulos et al. (24), which described the clinical presentation of a patient hospitalized following a suicide attempt who exhibited severe depressive symptoms, nihilistic and hypochondriacal delusions, and psychomotor retardation. The authors emphasize that affective and organic factors often co-occur in the background of the CS and they highlight the significance of suicide risk, refusal to eat, and severe impairment of reality testing. Morgado et al. (12) described CS in a schizophrenic patient, characterized by blunted emotions and increased psychomotor activity alongside a euthymic mood, but without pronounced depressive symptoms. In our case, CS presented in accordance with the complete form, with pronounced nihilistic delusions (25); however, the manner of its onset differed from the classically described gradual course.

In descriptions of the CS, the complete form refers to the dominant, relatively clear manifestation of nihilistic delusions, whereas in the incomplete form, the delusional content is embedded in a depressive mood, themes of guilt, hypochondriacal complaints, or hallucinatory experiences (26). The literature traditionally describes a gradually unfolding clinical course, in which prodromal, developmental, and chronic phases can be distinguished (27). Koreki et al. (28) described the case of a female patient with schizophrenia in whom, in addition to classic “I am already dead” Cotard delusions, nihilistic delusions directed at others also appeared: the patient believed that her doctor was also dead, which can be compared to the present case, where the patient firmly asserted that his wife had also died and that they would soon be reunited after death. The onset of CS has also been linked to patients with dementia and frontotemporal atrophy (25), where delusions were often accompanied by hypochondriacal complaints, refusal to eat, and progressive cognitive decline (18). These observations are consistent with our case, in which mild cognitive impairment and fronto-temporal atrophy were identified as underlying factors in the acute psychotic state, supporting the role of organic factors in the development of the Cotard phenomenon. In the report by Solimine et al. (14)—similar to the role played by aripiprazole monotherapy and supportive psychotherapy in the resolution of catatonic symptoms (Vörös & Tényi (29))—Cotard symptoms went into remission. This effect is also confirmed by De Berardis et al. (30). In our case, unlike the electroconvulsive therapy (ECT) emphasized in the literature and typically chosen as the first-line treatment, especially for symptoms with a depressive etiological background (4) (ECT) (7, 31), antipsychotic (risperidone 0.5 mg) (32) and antidepressant (mirtazapine 30 mg) pharmacotherapy proved to be effective (1). The case report by Ruminjo & Mekinulo (33) highlights that carefully selected, personalized medication alone can result in complete symptom remission.

Our case is similar in several respects to the case described by Debruyne et al. (1), in which an 88-year-old man with mild cognitive impairment was admitted to the hospital due to a severe depressive episode accompanied by Cotard-type nihilistic delusions: he was convinced that he had died and was anxious that he had not yet been buried. The delusions caused significant distress and made outpatient treatment impossible. Treatment with sertraline (50 mg) and risperidone (1 mg) resulted in complete remission of depressive symptoms and delusions; medication was continued thereafter. Leis et al. (34) and Barradas et al. (35) emphasize that, due to the rarity of the CS, knowledge is largely based on case studies, so etiological and therapeutic conclusions can only be drawn with caution; at the same time highlighting the frequent presence of structural brain abnormalities, the close association with depression, and the importance of early recognition as key considerations in assessing the risk of self-destructive behaviors and suicide, which our own case study also confirms.

## 5 Limitations

Due to the nature of this case study, the generalizability of the observations presented is limited.

Acute severe depression, recent delirium, somatic decompensation, sensory impairment, and advanced age may all have influenced MMPI-2 performance and profile interpretation. Consequently, MMPI-2 results were treated as supportive clinical information only and not as evidence of enduring personality structure or a primary psychotic disorder. Another limitation is that the diagnosis of delirium was based on clinical psychiatric and medical assessment rather than a standardized delirium rating instrument. Although the patient exhibited characteristic features of delirium, including acute onset, fluctuating course, impaired attention, disorientation, and altered awareness, neither the Confusion Assessment Method (CAM) nor the Delirium Rating Scale-Revised-98 (DRS-R-98) was administered during the acute episode.

## 6 Conclusion

Our observation supports the view that Cotard’s syndrome should not be interpreted as an independent nosological entity, but rather as a phenomenological manifestation arising from the interaction of affective, psychotic, delirious, neurocognitive, and somatic processes. In the present case, Cotard-type nihilistic delusions emerged in the context of severe depression and suicidal crisis, further complicated by somatic decompensation and a fluctuating delirious state on the background of neurocognitive vulnerability. This case underscores the importance of a comprehensive biopsychosocial approach, careful neurological and cognitive assessment, and timely multidisciplinary psychiatric care to optimize diagnostic accuracy, reduce suicide risk, and improve clinical outcomes.

## Data Availability

The raw data supporting the conclusions of this article will be made available by the authors, without undue reservation.
